# Use of Clesrovimab for Prevention of Severe Respiratory Syncytial Virus–Associated Lower Respiratory Tract Infections in Infants: Recommendations of the Advisory Committee on Immunization Practices — United States, 2025

**DOI:** 10.15585/mmwr.mm7432a3

**Published:** 2025-08-28

**Authors:** Danielle L. Moulia, Ruth Link-Gelles, Helen Y. Chu, Denise Jamieson, Oliver Brooks, Sarah Meyer, Eric S. Weintraub, David K. Shay, Mila M. Prill, Ebony S. Thomas, David Hutton, Ismael R. Ortega-Sanchez, Adam MacNeil, Meredith L. McMorrow, Jefferson M. Jones

**Affiliations:** ^1^Coronavirus and Other Respiratory Viruses Division, National Center for Immunization and Respiratory Diseases, CDC; ^2^University of Washington, Seattle, Washington; ^3^University of Iowa, Iowa City, Iowa; ^4^Watts Healthcare Corporation, Los Angeles, California; ^5^Division of Healthcare Quality Promotion, National Center for Emerging and Zoonotic Diseases, CDC; ^6^Immunization Services Division, National Center for Immunization and Respiratory Diseases, CDC; ^7^University of Michigan, Ann Arbor, Michigan.

**Summary**

**What is already known about this topic?**

To prevent respiratory syncytial virus (RSV)–associated lower respiratory tract infection (LRTI) in infants, since 2023, the Advisory Committee on Immunization Practices (ACIP) has recommended either 1) maternal RSV vaccination during pregnancy or 2) administration of nirsevimab, a long-acting RSV monoclonal antibody, to the infant.

**What is added by this report?**

On June 26, 2025, ACIP recommended clesrovimab, a newly licensed long-acting RSV monoclonal antibody, as an alternative to nirsevimab for infants aged <8 months born during or entering their first RSV season who did not receive protection through maternal RSV vaccination.

**What are the implications for public health practice?**

All infants should be protected against RSV LRTI through either maternal RSV vaccination or receipt of a long-acting RSV monoclonal antibody (clesrovimab or nirsevimab).

## Abstract

Before the introduction of universal respiratory syncytial virus (RSV) immunization recommendations for infants, RSV was the leading cause of hospitalization among infants in the United States. Since 2023, CDC’s Advisory Committee on Immunization Practices (ACIP) has recommended that all infants be protected against RSV-associated lower respiratory tract infection (LRTI) through either 1) maternal RSV vaccination during pregnancy (Abrysvo, Pfizer) or 2) administration of nirsevimab (Beyfortus, Sanofi and AstraZeneca), a long-acting RSV monoclonal antibody, to the infant. In June 2025, the Food and Drug Administration licensed clesrovimab (Enflonsia, Merck), a second long-acting RSV monoclonal antibody, for prevention of RSV-associated LRTI in infants. Since September 2024, the ACIP Maternal/Pediatric RSV Work Group has reviewed evidence regarding the safety and efficacy of clesrovimab use in infants. On June 26, 2025, ACIP recommended clesrovimab as a second long-acting monoclonal antibody product that could be used as an alternative to nirsevimab for prevention of RSV-associated LRTI among infants aged <8 months who are born during or entering their first RSV season and who are not protected through maternal RSV vaccination. All infants should be protected against RSV-associated LRTI through use of one of these three products (i.e., maternal RSV vaccination or administration of nirsevimab or clesrovimab to the infant). No one product is preferred; the choice should be guided by parent preference, product availability, and timing of the infant’s birth relative to the RSV season.

## Introduction

Before the introduction of universal respiratory syncytial virus (RSV) immunization recommendations for infants in 2023, RSV was the leading cause of hospitalization among U.S. infants (aged <12 months) (*1*): an estimated 2%–3% of infants aged <3 months were hospitalized for RSV each year (*2*). RSV led to an estimated 58,000–80,000 RSV-associated hospitalizations and 100–300 RSV-associated deaths annually among U.S. infants and children aged <5 years (*3*–*5*). The rate of RSV-associated hospitalization is highest during the first 6 months of life, peaking at age 1 month and decreasing with increasing age (*6*). Most infants hospitalized with RSV have no known risk factors for severe RSV. Thus, all infants are at risk for severe RSV disease (*7*).

Nirsevimab (Beyfortus, Sanofi, in collaboration with AstraZeneca), a long-acting* monoclonal antibody for prevention of RSV-associated lower respiratory tract infection (LRTI), was recommended by the Advisory Committee on Immunization Practices (ACIP) in August 2023; administration of RSV vaccine (Abrysvo, Pfizer) to pregnant women at 32–36 weeks’ gestation was recommended by ACIP in September 2023 (*8*). ACIP recommended that all infants be protected against RSV-associated LRTI through either 1) maternal RSV vaccination during pregnancy or 2) administration of a long-acting RSV monoclonal antibody (nirsevimab) to the infant. Use of both products was not recommended for most infants.

Data from real-world effectiveness studies estimate that nirsevimab and maternal RSV vaccination offer protection against approximately 70%–80% of RSV-associated hospitalizations among infants (*9*). During the 2024–2025 RSV season, the first full season when the two RSV prevention products were widely available, data from two national surveillance networks (the Respiratory Syncytial Virus Hospitalization Surveillance Network and the New Vaccine Surveillance Network) estimated that RSV-associated hospitalization rates among infants aged 0–7 months were 43% and 28% lower, respectively, compared with rates during pre–COVID-19 pandemic RSV seasons (*10*).

On June 9, 2025, the Food and Drug Administration (FDA) approved clesrovimab (Enflonsia, Merck), a second long-acting RSV monoclonal antibody, for prevention of RSV-associated LRTI among infants (*11*). Clesrovimab is administered as a single intramuscular injection before or during an infant’s first RSV season. On June 26, 2025, ACIP recommended clesrovimab as one of two long-acting monoclonal antibody options (i.e., clesrovimab or nirsevimab) for the prevention of RSV-associated LRTI in infants aged <8 months born during or entering their first RSV season who are not protected through maternal RSV vaccination. This report summarizes the evidence for clesrovimab and presents updated clinical guidance for protection against RSV-associated LRTI in infants.

## Methods

During September 2024–June 2025, the ACIP Maternal/Pediatric RSV Work Group met monthly to review evidence regarding the safety and efficacy of clesrovimab and determined the quality of this evidence using the Grading of Recommendations, Assessment, Development, and Evaluation (GRADE) approach (*12*). The Evidence to Recommendations (EtR) framework was used to guide the work group’s deliberations and develop RSV prevention product recommendations for infants and children (*13*); the work group provided input on voting language. The work group conclusions regarding evidence for the use of clesrovimab in infants were presented to ACIP at public meetings on October 16, 2024; April 24, 2025; and June 25, 2025 (*14*–*16*).

## Rationale and Evidence

### Efficacy and Safety

The ACIP Maternal/Pediatric RSV Work Group evaluated the safety and efficacy of clesrovimab using data from a single phase 2b/3 randomized, double-blind, placebo-controlled trial among infants born during or entering their first RSV season^†^ (*15**, **17*,*18*). A total of 3,614 healthy preterm and term infants born at ≥29 weeks’ gestation were randomized and dosed in a 2:1 ratio to receive clesrovimab (2,411) or placebo^§^ (1,203), with follow-up for efficacy in preventing RSV-associated medically attended LRTI and associated hospitalization through 150 days^¶^ after injection and for safety through 365 days after injection. The GRADE evidence profile and supporting EtR framework for the work group’s assessment of clesrovimab are available at GRADE | CDC and EtR Framework | CDC.

**Efficacy.** Efficacy** of clesrovimab in preventing RSV-associated medically attended LRTI^††^ through 150 days was 60.4% (95% CI = 44.1%–71.9%), with the outcome occurring in 60 (2.5%) of 2,398 participants in the clesrovimab group and 74 (6.2%) of 1,201 participants in the placebo group. Efficacy against RSV-associated LRTI hospitalization^§§^ was 90.9% (95% CI = 76.2%–96.5%), with the outcome occurring in five participants (0.2%) in the clesrovimab group and 27 (2.2%) participants in the placebo group (*17*,*18*).

**Safety.** The incidence of all-cause serious adverse events^¶¶^ through 1 year after injection was similar among infants who received clesrovimab (11.5%) compared with those who received placebo (12.4%) (relative risk = 0.93; 95% CI = 0.77–1.12). Most solicited adverse events were mild or moderate, with irritability and somnolence being the most common, and the frequencies of fever and injection-site reactions were similar between trial groups.

### Economic Analysis

The ACIP Maternal/Pediatric RSV Work Group also considered whether the use of clesrovimab for infants is a reasonable and efficient allocation of resources. University of Michigan and CDC staff members updated an economic analysis performed for nirsevimab (*19*), using the same model and inputs, except that the price of clesrovimab was based on a manufacturer-generated report, and clesrovimab efficacy inputs were based on clesrovimab clinical trial data (*15*). The cost-effectiveness model compared the use of clesrovimab in eligible infants with use of palivizumab (a monoclonal antibody that was previously recommended by the American Academy of Pediatrics for children with certain underlying medical conditions and requires monthly dosing)*** in eligible infants and no immunization in all other infants. This model found an incremental cost-effectiveness ratio of $105,000 per quality-adjusted life-year (QALY) gained from a societal perspective, with a range from cost-saving to approximately $213,000 per QALY gained in sensitivity analyses (*15*). The base case cost per dose was $457, and influential inputs included inpatient costs, clesrovimab cost per dose, and RSV-related QALYs lost (*15*).

## Recommendations for Use of Clesrovimab

On June 25, 2025, ACIP recommended clesrovimab as one of two long-acting monoclonal antibody options (i.e., as an alternative to nirsevimab), for the protection of infants aged <8 months born during or entering their first RSV season^†††^ who are not protected through maternal RSV vaccination. ACIP recommends that all infants be protected against RSV-associated LRTI through one of three product options: 1) maternal RSV vaccination during pregnancy; 2) infant receipt of the long-acting RSV monoclonal antibody, nirsevimab; or 3) infant receipt of the long-acting RSV monoclonal antibody, clesrovimab (Table). Health care providers should select which product to use based on parent preference, product availability, and timing relative to the RSV season, with an understanding of relative advantages and disadvantages (Box 1). Recommendations for nirsevimab and maternal vaccination have been previously published, and up-to-date clinical guidance for all three products have also been published (RSV Immunization Guidance for Infants and Young Children | CDC; RSV Vaccine Guidance for Pregnant Women | CDC).

**TABLE:** Products* to prevent respiratory syncytial virus–associated severe disease among infants aged <8 months born during or entering their first respiratory syncytial virus season — United States, 2025

Product	Population recommended to receive the product	Months when the product should be administered	Dosing information (by single IM injection)
RSV monoclonal antibody, clesrovimab (Enflonsia)^†^	Infants aged <8 mos	October–March^§,¶^	105 mg (0.7 mL) for all infants regardless of weight
RSV monoclonal antibody, nirsevimab (Beyfortus)^†^	Infants aged <8 mos	October–March^§,¶^	50 mg (0.5 mL) for infants weighing <11 lb (<5 kg) 100 mg (1 mL) for infants weighing ≥11 lb (≥5 kg)
Maternal RSV vaccine, RSVpreF (Abrysvo)	Pregnant women at 32–36 wks’ gestation	September–January**	0.5 mL

**Abbreviations**: ACIP = Advisory Committee on Immunization Practices; IM = intramuscular; RSV = respiratory syncytial virus.

* ACIP recommends that all infants be protected against RSV-associated lower respiratory tract infection through one of the three listed products.

^†^ A single dose of RSV monoclonal antibody is recommended for infants aged <8 months who are born during or entering their first RSV season (typically fall through spring in most of the continental United States) if 1) the mother did not receive RSV vaccine during pregnancy or 2) the mother’s RSV vaccination status is unknown, or 3) the infant was born ≤14 days after maternal RSV vaccination. Except in rare circumstances, administration of RSV monoclonal antibody is not indicated for most infants who are born ≥14 days after their mother received RSV vaccine.

^§^ Eligible infants born during the seasonal administration window for RSV monoclonal antibody products (October 1–March 31 in most of the continental United States) should receive RSV antibody within 1 week after birth, ideally during the birth hospitalization. Any eligible infant or young child born outside the seasonal administration window (April–September) who has not yet received a recommended dose should receive RSV antibody at the earliest opportunity beginning in October to optimize protection during the peak RSV season. For infants eligible for RSV antibody with prolonged hospitalizations shortly before or during the RSV season, providers may consider administering RSV antibody during the birth hospitalization to prevent health care–associated RSV disease. This decision should be based on clinical judgment considering the potential risks and benefits.

^¶^ RSV monoclonal antibody should be administered during October through the end of March in most of the continental United States. However, ACIP recommendations on the timing of RSV antibody administration are intentionally flexible to optimize patient access, including reimbursement. For example, In Alaska RSV circulation patterns are less predictable, and the duration of the RSV season is often longer than the national average. Tropical climates might also have RSV circulation patterns that differ from most of the continental United States or that are unpredictable. In addition, because the timing of RSV activity varies geographically in other regions of the United States, public health authorities may elect to provide revised guidance regarding the timing of RSV antibody administration based on local surveillance data and feasibility of implementation (i.e., extending or shortening the recommended administration period of October–March). Public health authorities should consider the advantages and disadvantages of modifying the timing of administration. Providers, including regional medical centers and health systems, should consult with state or territorial health departments before systematically modifying the recommended months for RSV antibody administration for their eligible patient populations.

** In areas where the timing of the RSV season is less predictable, providers should follow state or territorial guidance on timing of maternal RSV vaccination. In addition, state and territorial health departments can consider modifying the timing of administration based on historical seasonality data and should consider the potential advantages and disadvantages of any modifications. Providers, including regional medical centers and health systems, should consult with state or territorial health departments before systematically modifying the recommended months for maternal vaccination for their eligible patient populations.

BOX 1 **Advantages and disadvantages of respiratory syncytial virus maternal vaccination and respiratory syncytial virus long-acting monoclonal antibodies (clesrovimab or nirsevimab) for protection against respiratory syncytial virus–associated severe disease among infants aged <8 months born during or entering their first respiratory syncytial virus season — United States, 2025**

**Maternal RSV Vaccine (RSVpreF)**

**Advantages**

- Provides protection immediately after birth
- Might be more resistant to potential RSV mutation*

**Disadvantages**

- Protection potentially reduced if fewer antibodies are produced or are transferred from mother to infant (e.g., mother is immunocompromised, infant is born soon after vaccination, or infant has prematurity)
- Potential risk for hypertensive disorders of pregnancy^†^

**RSV Monoclonal Antibodies**

**Advantages**

- Protection might last longer
- Ensures infant receives antibodies directly rather than relying on transplacental transfer
- No risk for adverse pregnancy outcomes

**Disadvantages**

- Requires infant to receive an injection
- Product might not be available^§^

**Abbreviations**: RSV = respiratory syncytial virus; RSVpreF = maternal RSV vaccine.

* RSVpreF vaccination results in a polyclonal antibody response, which is expected to be more resistant to potential mutations in the RSV F protein than is a monoclonal RSV antibody.

^†^ In a 2025 Vaccine Safety Datalink matched cohort study of vaccinated and unvaccinated pregnant women (13,474 matched pairs), an association between RSVpreF vaccination and hypertensive disorder of pregnancy (adjusted odds ratio = 1.09 [95% CI = 1.03–1.15]) was observed. RSVpreF Vaccine Safety 2023–24 Respiratory Season | Health Partners Institute

^§^ RSV monoclonal antibody products might not be available in all birthing hospitals and outpatient clinics.

### Routine Administration of RSV Monoclonal Antibodies

For infants aged <8 months born during or entering their first RSV season, the recommendations for nirsevimab and clesrovimab are the same, with the exception that the dose of clesrovimab (105 mg) is the same for all infants, whereas the nirsevimab dose differs depending on the infant’s weight.^§§§^ In the following recommendations, RSV antibody refers to either nirsevimab or clesrovimab. A dose of RSV antibody is recommended for all eligible infants (i.e., those aged <8 months^¶¶¶^ born during or entering their first RSV season, typically fall through spring in the continental United States) if 1) the mother did not receive RSV vaccine during pregnancy, 2) the mother’s RSV vaccination status is unknown, or 3) the infant was born ≤14 days after maternal RSV vaccination. Except in rare circumstances, RSV antibody is not indicated for most infants who are born ≥14 days after their mother received RSV vaccine.**** RSV antibodies and routine childhood vaccines may be administered during the same visit.

### Timing of RSV Monoclonal Antibody Administration

**Administration to general infant population. **Eligible infants born during the seasonal administration window for RSV antibody (October 1–March 31 in most of the continental United States) should receive the RSV antibody dose within 1 week after birth, ideally during the birth hospitalization. Eligible infants born outside the seasonal administration window (i.e., during April–September) who have not yet received a recommended dose should receive RSV antibody at the earliest opportunity beginning in October to optimize protection during the peak of the RSV season.

ACIP recommendations on the timing of RSV antibody administration are intentionally flexible to improve patient access, including reimbursement. For example, in Alaska, RSV circulation patterns are less predictable, and the duration of the RSV season is often longer than the national average. Tropical climates^††††^ might also have RSV circulation patterns that differ from those in most of the continental United States or that are unpredictable.

In addition, because the timing of RSV activity varies geographically in other regions of the United States (*20*), public health authorities may provide revised guidance regarding the timing of RSV antibody administration based on local surveillance data and feasibility of implementation (i.e., extending or shortening the recommended administration period of October–March).^§§§§^ Public health authorities should consider the potential advantages and disadvantages of modifying the timing of administration (Box 2). Providers, including regional medical centers and health systems, should consult with state or territorial health departments^¶¶¶¶^ before systematically modifying the recommended months for RSV antibody administration for their eligible patient populations.*****

BOX 2 **Potential advantages and disadvantages of modifying timing of administration of maternal respiratory syncytial virus vaccine and respiratory syncytial virus long-acting monoclonal antibodies (clesrovimab or nirsevimab) — United States, 2025**

**Starting Administration of Maternal Vaccine Before September or RSV Monoclonal Antibodies Before October**

**Advantages**

- **RSVpreF maternal vaccine:** Can provide protection to infants born before October and for infants who might experience a delay in receipt of RSV antibody
- **RSV monoclonal antibody**: Can provide more time for infants to receive RSV antibody before start of the RSV season
- Potentially useful for jurisdictions with early RSV seasonality

**Disadvantage**

- Maximum protection is expected shortly after administration (for infant RSV monoclonal antibody) or birth (for RSVpreF maternal vaccine). Protection wanes over time, although the rate of decrease is unknown. Therefore, infants who receive RSV monoclonal antibody in September or who are born to a mother who was vaccinated in August, might be less protected during the peak of the RSV season and toward the end of the RSV season.

**Extending Duration of Administration of Maternal RSV Vaccine Past January or RSV Monoclonal Antibodies Past March**

**Advantage**

- Either product type could protect infants during their first months of life when they are at highest risk for severe disease.

**Disadvantages**

- The risk for RSV exposure and infection toward the end of the RSV season might be low.
- **RSVpreF maternal vaccine:** A woman vaccinated at 32–36 weeks’ gestation during February or March could give birth to the infant in April or May, and that infant would generally not be recommended to receive an RSV monoclonal antibody* dose in October because most infants born to vaccinated mothers are not recommended to receive RSV monoclonal antibody. Administering a dose in October instead of vaccinating the mother could provide protection for an entire RSV season.
- **RSV monoclonal antibody**: Most infants born to unvaccinated mothers are recommended to receive only 1 dose of an RSV monoclonal antibody. Therefore, most infants who receive RSV antibody in April would not be recommended to receive a dose in October. Administering the dose in October instead of April could provide protection for an entire RSV season.

**Abbreviations:** RSV = respiratory syncytial virus; RSVpreF = maternal RSV vaccine.

* Infants born <14 days after maternal vaccination should receive RSV monoclonal antibody. RSV monoclonal antibody may also be considered for infants born to vaccinated mothers in rare circumstances when, based on the clinical judgment of the health care provider, the potential incremental benefit of administration is warranted. These situations include but are not limited to 1) infants born to mothers who might not mount an adequate immune response to vaccination (e.g., mothers with immunocompromising conditions); 2) infants born to mothers who have conditions associated with reduced transplacental antibody transfer (e.g., mothers living with HIV infection); 3) infants who undergo procedures leading to loss of maternal antibodies (e.g., cardiopulmonary bypass, extracorporeal membrane oxygenation, or exchange transfusion); and 4) infants at substantially increased risk for severe RSV disease (e.g., hemodynamically significant congenital heart disease or intensive care unit admission with oxygen requirement at discharge).

**Administration to hospitalized infants.** Health care–associated RSV disease occurs; however, the incidence is unknown (*21*,*22*). Standard and contact infection control practices are indicated to decrease health care–associated RSV disease (*23*). Safety data for use of RSV antibody in infants with a postmenstrual age (gestational age at birth plus chronologic age) of <32 weeks or who weigh <3.5 lb (<1.6 kg) are limited (Summary of Product Characteristics | Beyfortus | INN-nirsevimab) (*24*). To prevent health care–associated RSV disease, providers may consider administering RSV antibody to eligible hospitalized infants during their hospitalization. This decision should be based on clinical judgment, considering the potential risks and benefits, as well as local RSV activity.

### Precautions and Contraindications

Clesrovimab is contraindicated for and should not be administered to persons with a history of severe allergic reaction, such as anaphylaxis, to any product component. Components can be found in the package inserts for clesrovimab: Clesrovimab Label | FDA.

### Reporting of Adverse Events

Adverse events might occur after administration of an RSV antibody. Reporting of any clinically significant adverse event is encouraged, regardless of whether a causal relationship with the product is certain.

Adverse events after administration of an RSV antibody alone should be reported to FDA’s MedWatch system online (The FDA Safety Information and Adverse Event Reporting Program | Medwatch), by fax, by mail, or by calling 800-332-1088.^†††††^

Adverse events after coadministration of an RSV antibody with a vaccine should be reported to the Vaccine Adverse Event Reporting System (VAERS) online at Vaccine Adverse Event Reporting System (VAERS), by fax, by mail, or by calling 800-822-7967. The report should specify that the patient received nirsevimab or clesrovimab.^§§§§§^ If an adverse event occurs after coadministration of an RSV antibody with a vaccine and is reported to VAERS, a duplicate report to MedWatch is not necessary.

## Footnotes

* Long-acting monoclonal antibodies for RSV require only 1 dose for protection during an RSV season. This differs from palivizumab, a monoclonal antibody that requires regular dosing throughout the season.

† Clesrovimab was also evaluated in a phase 3 trial in infants and children with increased risk for severe RSV disease who were randomized to receive either clesrovimab or palivizumab. This trial was not placebo-controlled and was therefore excluded from the ACIP Maternal/Pediatric RSV Work Group’s review of evidence for GRADE. This trial has not yet been completed as of the publication of this report. Preliminary data presented to ACIP on October 23, 2024, indicated that adverse events were generally comparable in the palivizumab and clesrovimab groups (Clesrovimab (MK-1654): Pediatric Clinical Program). The incidence of RSV-associated medically attended LRTI and hospitalization were similar between the palivizumab and clesrovimab groups through 6 months after injection. The concentration of clesrovimab as measured by an area under the curve through 150 days after injection were similar in the clesrovimab groups of infants and children with increased risk for severe RSV disease in this phase 3 trial and the healthy infants and children in the phase 2b/3 trial. A 105-mg dose of clesrovimab was given in both trials, and the pharmacokinetic bridging data suggest that clesrovimab would similarly protect infants and children with and without increased risk for severe RSV disease using the same dose.

§ Placebo was 0.9% sodium chloride.

¶ The phase 2b/3 trial’s primary outcome was RSV-associated medically attended LRTI through 150 days. RSV-associated medically attended LRTI associated with hospitalization was a tertiary outcome. Additional outcomes included RSV-associated medically attended LRTI and associated hospitalization through 180 days.

** Efficacy was defined as (1 − relative risk) × 100, and the relative risk was estimated comparing the incidence of the outcome in the clesrovimab trial group divided by the incidence in the placebo group, using a modified Poisson regression approach with robust variance estimation.

†† RSV-associated medically attended LRTI was defined by the presence of the following in an infant seen in an outpatient or inpatient clinical setting: cough or difficulty breathing and one or more indicators of LRTI or severity (wheezing, chest wall in-drawing or retractions, rales or crackles, hypoxemia, tachypnea, and dehydration associated with respiratory signs or symptoms), and a nasopharyngeal sample that tested positive for RSV by reverse transcription–polymerase chain reaction (RT-PCR).

§§ RSV-associated LRTI hospitalization was defined as the presence of the following in an infant seen in an inpatient clinical setting: cough or difficulty breathing and one or more indicators of LRTI (wheezing, rhonchi, rales, or crackles), one or more indicators of severity (chest wall indrawing or retractions, hypoxemia, tachypnea, and dehydration associated with respiratory signs or symptoms), and a nasopharyngeal sample that tested positive for RSV by RT-PCR.

¶¶ Serious adverse events are defined as any medical occurrence that results in death, is life-threatening, requires inpatient hospitalization or prolongation of existing hospitalization, or results in persistent disability or incapacity.

*** Before licensure of nirsevimab, the only FDA-approved product to prevent severe RSV disease among infants and young children was palivizumab, another monoclonal antibody. Before the licensure and recommendation of nirsevimab, the American Academy of Pediatrics (AAP) recommended palivizumab for infants and children with certain underlying medical conditions (accounting for <5% of all infants and children); its use is further limited by high cost and the requirement for monthly dosing. AAP no longer routinely recommends palivizumab, and palivizumab will no longer be available in the United States as of December 31, 2025. Respiratory Syncytial Virus | Red Book: 2024–2027 Report of the Committee on Infectious Diseases | Red Book Online | American Academy of Pediatrics; SYNAGIS (palivizumab) | Parent & Caregiver Website

††† Nirsevimab is the only product recommended by CDC for infants and children aged 8–19 months at high risk for RSV-associated LRTI entering their second RSV season.

§§§ The dose of nirsevimab is 50 mg for infants weighing <11 lb (<5 kg) and 100 mg for infants weighing ≥11 lb (≥5 kg).

¶¶¶ The infant or child’s age on the day of administration should be used to determine whether they are eligible for immunization.

**** If an infant is born <14 days after maternal vaccination the infant should receive RSV monoclonal antibody. RSV monoclonal antibody may also be considered for infants born to vaccinated mothers in rare circumstances when, based on the clinical judgment of the health care provider, the potential incremental benefit of administration is warranted. These situations include but are not limited to infants born to mothers who might not mount an adequate immune response to vaccination (e.g., mothers with immunocompromising conditions); infants born to mothers who have conditions associated with reduced transplacental antibody transfer (e.g., mothers living with HIV infection); infants who undergo procedures leading to loss of maternal antibodies (e.g., cardiopulmonary bypass, extracorporeal membrane oxygenation, or exchange transfusion); and infants at substantially increased risk for severe RSV disease (e.g., hemodynamically significant congenital heart disease or intensive care unit admission with oxygen requirement at discharge).

†††† Locations with tropical climates include but are not limited to southern Florida, Guam, Hawaii, Puerto Rico, U.S.-affiliated Pacific Islands, and the U.S. Virgin Islands.

§§§§ In areas with clear increases in RSV transmission before October, administration before October can be considered. In areas with high RSV transmission through the end of March, administering to newborns beyond the end of March can be considered. Beginning administration after October or ending administration before March can also be considered.

¶¶¶¶ Consultation with health departments will ensure that RSV monoclonal antibody will be available for use when the months of administration are modified. Consultation with health departments by health care systems will also ensure that health care providers within the system are receiving consistent recommendations on the timing of RSV antibody administration.

***** Health care providers may use clinical judgement in determining when to give RSV antibody outside the months of October–March. Special circumstances might also need to be considered, such as travel to areas with increased RSV activity or concerns that the infant or child might not return for a visit when nirsevimab should ideally be administered. When health care providers are using clinical judgment to adjust the timing of RSV antibody for individual patients, consultation with state or territorial health care providers is not needed.

††††† Adverse events can be reported to MedWatch because FDA has classified clesrovimab and nirsevimab as drugs.

§§§§§ Information about any prescriptions, over-the-counter medications, dietary supplements, or herbal remedies being take at the time of vaccination is solicited in section 9 of the VAERS report.

## References

[1] Suh M, Movva N, Jiang X, Respiratory syncytial virus is the leading cause of United States infant hospitalizations, 2009–2019: a study of the national (nationwide) inpatient sample. J Infect Dis 2022;226(Suppl 2):S154–63. 10.1093/infdis/jiac120 35968878 PMC9377046

[2] McMorrow ML, Moline HL, Toepfer AP, Respiratory syncytial virus–associated hospitalizations in children <5 years: 2016–2022. Pediatrics 2024;154:e2023065623. 10.1542/peds.2023-065623 38841769 PMC11890375

[3] Hall CB, Weinberg GA, Iwane MK, The burden of respiratory syncytial virus infection in young children. N Engl J Med 2009;360:588–98. 10.1056/NEJMoa0804877 19196675 PMC4829966

[4] McLaughlin JM, Khan F, Schmitt HJ, Respiratory syncytial virus–associated hospitalization rates among US infants: a systematic review and meta-analysis. J Infect Dis 2022;225:1100–11. 10.1093/infdis/jiaa752 33346360 PMC8921994

[5] Hansen CL, Chaves SS, Demont C, Viboud C. Mortality associated with influenza and respiratory syncytial virus in the US, 1999–2018. JAMA Netw Open 2022;5:e220527. 10.1001/jamanetworkopen.2022.0527 35226079 PMC8886548

[6] Curns AT, Rha B, Lively JY, Respiratory syncytial virus–associated hospitalizations among children <5 years old: 2016 to 2020. Pediatrics 2024;153:e2023062574. 10.1542/peds.2023-062574 38298053

[7] Hall CB, Weinberg GA, Blumkin AK, Respiratory syncytial virus–associated hospitalizations among children less than 24 months of age. Pediatrics 2013;132:e341–8. 10.1542/peds.2013-0303 23878043

[8] Fleming-Dutra KE, Jones JM, Roper LE, Use of the Pfizer respiratory syncytial virus vaccine during pregnancy for the prevention of respiratory syncytial virus–associated lower respiratory tract disease in infants: recommendations of the Advisory Committee on Immunization Practices—United States, 2023. MMWR Morb Mortal Wkly Rep 2023;72:1115–22. 10.15585/mmwr.mm7241e1 37824423 PMC10578951

[9] MacNeil A. CDC. Effectiveness and impact of RSV prevention products in infants during the 2024–2025 RSV season. [Presentation slides]. Presented at the Advisory Committee on Immunization Practices meeting, Atlanta, GA; June 26, 2025. https://www.cdc.gov/acip/downloads/slides-2025-06-25-26/03-MacNeil-Mat-Peds-RSV-508.pdf

[10] Patton ME, Moline HL, Whitaker M, Interim evaluation of respiratory syncytial virus hospitalization rates among infants and young children after introduction of respiratory syncytial virus prevention products—United States, October 2024–February 2025. MMWR Morb Mortal Wkly Rep 2025;74:273–81. 10.15585/mmwr.mm7416a1 40338822 PMC12061057

[11] Food and Drug Administration. Enflonsia (clesrovimab-cfof) [Package insert]. Silver Spring, MD: US Department of Health and Human Services, Food and Drug Administration; 2025. https://www.accessdata.fda.gov/drugsatfda_docs/label/2025/761432s000lbl.pdf

[12] CDC. Grading of recommendations, assessment, development, and evaluation (GRADE): clesrovimab. Atlanta, GA: US Department of Health and Human Services, CDC; 2023. https://www.cdc.gov/acip/grade/clesrovimab-infants.html

[13] CDC. ACIP evidence to recommendations for use of clesrovimab in infants born during RSV season and entering their first RSV season. Atlanta, GA: US Department of Health and Human Services, CDC; 2023. https://www.cdc.gov/acip/evidence-to-recommendations/clesrovimab-infants.html

[14] Moulia DL; CDC. Maternal and pediatric RSV work group interpretations and next steps. [Presentation slides]. Presented at the Advisory Committee on Immunization Practices meeting, Atlanta, GA; October 23, 2024. https://www.cdc.gov/acip/downloads/slides-2024-10-23-24/04-RSV-Mat-Peds-Moulia-508.pdf

[15] Moulia DL; CDC. Evidence to recommendations framework: clesrovimab. [Presentation slides]. Presented at the Advisory Committee on Immunization Practices meeting, Atlanta, GA; April 16, 2025. https://www.cdc.gov/acip/downloads/slides-2025-04-15-16/02-Moulia-maternal-peds-RSV-508.pdf

[16] MacNeil A; CDC. Updates on evidence to recommendations framework: clesrovimab. [Presentation slides]. Presented at the Advisory Committee on Immunization Practices meeting, Atlanta, GA; June 26, 2025. https://www.cdc.gov/acip/downloads/slides-2025-06-25-26/05-MacNeil-Mat-Peds-RSV-508.pdf

[17] Sinha A. Merck. Clesrovimab (MK-1654): Pediatric clinical program. [Presentation slides]. Presented at the Advisory Committee on Immunization Practices meeting, Atlanta, GA; October 23, 2024. https://www.cdc.gov/acip/downloads/slides-2024-10-23-24/02-RSV-Mat-Peds-Sinha-508.pdf

[18] Food and Drug Administration, Center for Drug Evaluation and Research. Integrated review of clesrovimab application. Silver Spring, MD: US Department of Health and Human Services, Food and Drug Administration; 2025. https://www.accessdata.fda.gov/drugsatfda_docs/nda/2025/761432Orig1s000IntegratedR.pdf

[19] Hutton DW, Prosser LA, Rose AM, Cost-effectiveness of nirsevimab for respiratory syncytial virus in infants and young children. Pediatrics 2024;154:e2024066461. 10.1542/peds.2024-066461 39582316

[20] Hamid S, Winn A, Parikh R, Seasonality of respiratory syncytial virus—United States, 2017–2023. MMWR Morb Mortal Wkly Rep 2023;72:355–61. 10.15585/mmwr.mm7214a1 37022977 PMC10078848

[21] Gettler EB, Talbot HK, Zhu Y, Respiratory syncytial virus: an under-recognized healthcare-associated infection. Infect Control Hosp Epidemiol 2025;46:1–5; Epub May 16, 2025. 10.1017/ice.2025.88 40376828 PMC12169951

[22] Langley JM, LeBlanc JC, Wang EE, Nosocomial respiratory syncytial virus infection in Canadian pediatric hospitals: a Pediatric Investigators Collaborative Network on Infections in Canada Study. Pediatrics 1997;100:943–6. 10.1542/peds.100.6.943 9374561

[23] Kimberlin DW, Banerjee R, Barnett ED, eds. Respiratory Syncytial Virus [Section 3]. In: Red Book: 2024–2027 Report of the Committee on Infectious Diseases, 32nd ed.; 2024. https://publications.aap.org/redbook/book/755/Red-Book-2024-2027-Report-of-the-Committee-on?autologincheck=redirected

[24] MacNeil A. Work group considerations and clinical considerations for clesrovimab. [Presentation slides]. Presented at the Advisory Committee on Immunization Practices meeting, Atlanta, GA; June 26, 2025. https://www.cdc.gov/acip/downloads/slides2025-06-25-26/06-MacNeil-Mat-Peds-RSV-508.pdf

